# Waist Circumference Is More Closely Associated with Hypogonadism than Is Hyperglycemia, Independent of BMI in Middle-Aged Men

**DOI:** 10.1155/2021/1347588

**Published:** 2021-12-20

**Authors:** Po-Sheng Hsu, Chia-Lien Hung, Shih-Kai Tu, Hsin-Hung Chen, Deng-Ho Yang, Chun-Cheng Liao

**Affiliations:** ^1^Department of Family Medicine, Taichung Armed Forces General Hospital, Taichung, Taiwan; ^2^Department of Medical Education and Research, Taichung Armed Forces General Hospital, Taichung, Taiwan; ^3^School of Medicine, National Defense Medical Center, Taipei, Taiwan; ^4^Division of Endocrinology and Metabolism, Department of Internal Medicine, Asia University Hospital, Taichung, Taiwan; ^5^School of Medicine, Chung Shan Medical University, Taichung, Taiwan; ^6^Chung Sheng Clinic, Nantou, Taiwan; ^7^Division of Rheumatology/Immunology/Allergy, Department of Internal Medicine, Taichung Armed Forces General Hospital, Taichung, Taiwan

## Abstract

**Introduction:**

To evaluate whether waist circumference (WC) or hyperglycemia is more closely associated with hypogonadism in middle-aged men. *Research Design and Methods*. This cross-sectional study analyzed male participants under 65 years old from the MJ Health Screening Center in Taiwan from 2007 to 2016. Basic patient characteristics with relevant parameters were obtained. We used the chi-square test to perform a correlation analysis for HbA1c and WC between participants with and without hypogonadism. A one-way ANOVA with post hoc Scheffe's method was applied to compare the mean testosterone (T) among the HbAlc and WC groups (normal blood sugar with normal WC (NBSNW), abnormal blood sugar with normal WC (ABSNW), normal blood sugar with abnormal WC (NBSAW), and abnormal blood sugar with abnormal waist circumference (ABSAW)).

**Results:**

The 5,680 participants were divided into two groups based on the presence (*n* = 599) or absence of hypogonadism (*n* = 5,081), which was defined as total testosterone (TT) < 300 ng/dL. The mean TT of group NBSAW (443.71 ± 220.59 ng/dl) was significantly lower than that of group ABSNW (506.64 ± 191.08 ng/dl, *p* < 0.001). Moreover, the mean TT of group ABSAW (398.89 ± 146.24 ng/dl) was significantly lower than that of group ABSNW (506.64 ± 191.08 ng/dl, *p* < 0.001). The ORs after adjusting for BMI, TG, HDL, SBP, and DBP were statistically significant when comparing NBSAW vs. NBSNW (OR = 2.846; 95%CI = 2.266–3.575; *p* < 0.001), ABSNW vs. NDNW (OR = 1.693; 95%CI = 1.309–2.189; *p* < 0.001), and ABSAW vs. NBSNW (OR = 4.613; 95%CI = 3.634–5.856; *p* < 0.001).

**Conclusion:**

The current study showed that WC should be the risk factor that is more closely associated with hypogonadism than hyperglycemia in middle-aged men.

## 1. Introduction

Testosterone (T) plays an important role in the maintenance and development of male reproductive and sexual functions, muscle and bone health, body composition, and cognitive functions [1]. T also maintains psychological features, including mood, vitality, and sexual interest, which contribute to quality of life [2]. Male hypogonadism is the failure of the testes to produce T because of a distant (pituitary/hypothalamus) or local (testicular) deficiency [3, 4]. Hypogonadism is typically diagnosed based on the signs and symptoms associated with low T, followed by biochemical confirmation. One study based on the general United States population revealed that hypogonadism is estimated to affect between 2.1% and 12.8% of adult males, and more than 4 million American men are diagnosed with symptomatic androgen deficiency [5]. The hypogonadism in Males (HIS) study in 2006 revealed a 38.7% crude prevalence of hypogonadism (total testosterone (TT) < 300 ng/dL) in men aged >45 years [6].

Previous cross-sectional studies reported that type 2 diabetes is associated with lower TT levels [7, 8]. Over the last three decades, it has become apparent that T plays an important role in glucose homeostasis in men. Specifically, low levels of T are associated with or can predict the development of metabolic syndrome and diabetes mellitus [9]. Waist circumference (WC) is also considered a hypogonadism risk factor, which may contribute to sexual dysfunction, voiding symptoms, and other psychosomatic problems [10]. A European article revealed that the lowest levels of total and free T were observed in men with a relatively high WC despite relatively low overall obesity (measured by body mass index (BMI)), which suggests that WC should be the preferred anthropometric measurement for predicting endogenous T levels [11].

Many studies have indicated that male hypogonadism is associated with a higher risk of metabolic syndrome [12], incident cardiovascular disease [13], and erectile dysfunction [14]. These conditions may also develop in people with abnormal HbA1c levels and increased WC because both factors are inversely associated with TT [7, 15].

Based on previously published research, we are unable to determine whether WC or hyperglycemia has a greater association with hypogonadism.

Therefore, this study is aimed at evaluating the risk factors of hypogonadism among middle-aged Taiwanese men to determine whether hyperglycemia or WC has a greater impact on the risk of hypogonadism. This study is aimed at identifying a more convenient and specific parameter for the easy and early detection of hypogonadism.

## 2. Materials and Methods

### 2.1. Study Population

This cross-sectional study retrieved data from a large private health examination institute (MJ Health Screening Center) in Taiwan from 2007 to 2016. Given that this institute provides self-paid health examination services in major districts in Taiwan with a general payment of approximately 200-730 USD (about 17 USD, specifically on serum T), the demographic characteristics of the included patients were believed to be similar to those of the general Taiwanese population [12].

Overall, 5,680 male participants, regardless of any underlying disease, were identified during the designated period. Participants with insufficient data for evaluating the status of metabolic syndrome, serum T, or were female, as well as males > 65 years, were excluded.

All participants were divided into groups according to the presence or absence of hypogonadism. The cutoff point for T was set according to HIS study from 2006 [6], which defined hypogonadism as TT < 300 ng/dL. There were 5,081 participants without hypogonadism and 599 participants with hypogonadism. All data used in this research were authorized by and received from the MJ Health Research Foundation (Authorization Code: MJHRF2019016A). This study was approved by the Tri-Service General Hospital Institutional Review Board (number: A202005160) and was conducted in accordance with the principles stated in the Declaration of Helsinki. Any interpretations or conclusions presented in this paper do not represent the views of the MJ Health Research Foundation. All participants in the research gave written informed consent before the health examination to authorize the data analysis. Personal identification data were removed from the MJ Health Research Foundation, so the participants remained anonymous throughout the entire research process. The details of the study population and data collection are described and reported elsewhere [12].

### 2.2. Definition of Baseline Parameters

Clinical data and baseline parameters, including BMI, blood pressure, WC, high-density lipoprotein (HDL), triglycerides (TG), and fasting blood sugar (FBS), were collected. The chemiluminescent microparticle immunoassay (ARCHITECT i2000) was used for measuring T, the Homogeneous Direct method (TOSHIBA C8000) was used for HDL cholesterol, the GPO-POD-ESPT method (TOSHIBA C8000) was used for TG, and the HK.G-6-PD.NADP method (TOSHIBA C8000) was used for FBS.

The waistline was measured at the middle of the top of the hip bone and the bottom of the ribs, without clothing that might interfere with the measurement.

Hemoglobin A1C (HbA1c; glycated hemoglobin) is the most widely used clinical test to estimate mean blood glucose levels. It is used to diagnose diabetes and to monitor the efficacy of medical treatment. HbA1c has become increasingly use for diabetes screening among physicians due to its convenience of sampling, suitability as a chronic hyperglycemia index, low intraindividual variability, and propitious assay standardization. In 2010, the American Diabetes Association (ADA) suggested that HbA1c values of 5.7%–6.4% could be used to establish a diagnosis of prediabetes, whereas a value of >6.5% indicates diabetes [16].

### 2.3. Subgroup Analysis

We divided the patients into subgroups according to their HbA1c and WC status as follows: NBSNW, normal blood sugar (HbA1c < 5.7%) with normal WC (WC ≤ 90 cm); NBSAW, normal blood sugar (HbA1c < 5.7%) with abnormal WC (WC > 90 cm); ABSNW, abnormal blood sugar (HbA1c ≥ 5.7%) with normal WC (WC ≤ 90 cm); and ABSAW: abnormal blood sugar (HbA1c ≥ 5.7%) with abnormal WC (WC > 90 cm).

### 2.4. Statistical Analysis

Comparisons between participants with and without hypogonadism were performed using independent *T* tests. Chi-square test was used to perform a correlation analysis for HbA1c and WC between participants with and without hypogonadism. One-way ANOVA with the post hoc Scheffe's method was applied to compare the mean *T* values among the HbA1c and WC groups (NBSNW, ABSNW, NBSAW, and ABSAW). Finally, a regression model was applied to analyze the odds ratios (ORs) for the presence or absence of hypogonadism in the HbA1c and WC groups after adjusting for BMI, TG, HDL, systolic blood pressure (SBP), and diastolic blood pressure (DBP). Two-sided *p* < 0.05 indicated statistical significance. SPSS 26.0 was used for the statistical analysis.

## 3. Results

### 3.1. Characteristics of the Participants with and without Hypogonadism

Basic patient characteristics are summarized in Table 1. After determining the mean values of each parameter, significant differences in all variables except for age were observed between the groups with and without hypogonadism. The hypogonadism group had significantly lower serum T levels, higher BMI, larger WC, higher HbA1c, higher TG, lower HDL, higher blood pressure, and higher FBS compared with the group without hypogonadism.

### 3.2. Correlation Analysis of HbA1c and WC in Patients with and without Hypogonadism

We used the chi-square test to perform a correlation analysis for HbA1c and WC between the groups with and without hypogonadism (Table 2). The results revealed significant differences in the correlation analysis by HbA1c alone, WC alone, and HbA1c and WC together (*p* < 0.001).

### 3.3. Logistic Regression Analysis Stratified by WC or HbA1c

We used the logistic regression analysis for analyzing the relationship between with or without hypogonadism in the HbA1c and WC groups after adjusting for BMI, TG, HDL, systolic blood pressure (SBP), and diastolic blood pressure (DBP) (Table 3).

If we use logistic regression analysis for HbA1c stratified by WC, the OR of hypogonadism in the normal waist circumference group is 1.433 (*p* = 0.010); the OR of hypogonadism in the abnormal waist circumference group is 1.473 (*p* = 0.008). If we use logistic regression analysis for WC stratified by HbA1c, the OR of hypogonadism in the normal HbA1c group is 1.591 (*p* < 0.001); the OR of hypogonadism in the abnormal HbA1c group is 1.837 (*p* = 0.001).

### 3.4. One-Way ANOVA Analysis

**Figure 1:**
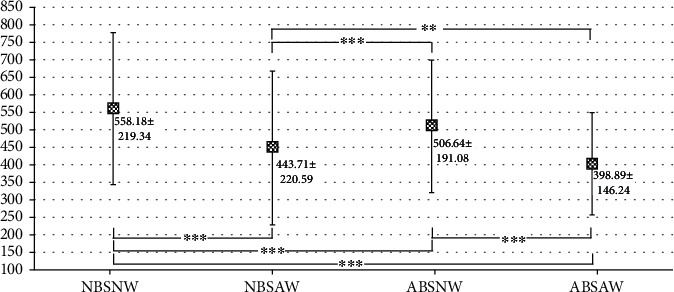
Comparisons between the HbA1c and WC groups were performed using an ANOVA test, followed by Scheffe's posttest. ^∗^*p* < 0.05; ^∗∗^*p* < 0.01; ^∗∗∗^*p* < 0.001.

Figure 1 shows the statistical differences among the groups. The mean TT of group NBSNW was significantly higher than that of every other group (558.18 ± 219.34 ng/dl, *p* < 0.01). The mean TT of group NBSAW (443.71 ± 220.59 ng/dl) was significantly lower than that of group ABSNW (506.64 ± 191.08 ng/dl, *p* < 0.001) and was significantly higher than that of group ABSAW (398.89 ± 146.24 ng/dl, *p* < 0.01). The mean TT of group ABSAW (398.89 ± 146.24 ng/dl) was significantly lower than that of group ABSNW (506.64 ± 191.08 ng/dl, *p* < 0.001).

### 3.5. Logistic Regression Analysis for the Odd Ratios of the Groups with and without Hypogonadism

**Figure 2:**
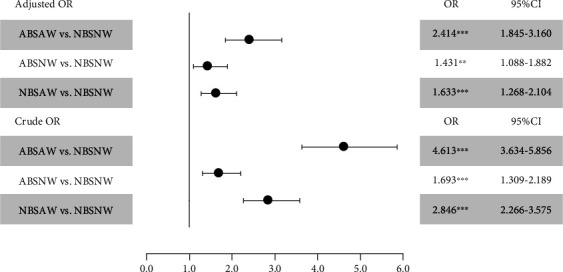
Odds ratios for groups with or without hypogonadism by logistic regression.

After adjusting for BMI, TG, HDL, SBP, and DBP in the multivariable logistic regression (Figure 2), the adjusted ORs highlighted statistically significant differences in NBSAW vs. NBSNW (OR = 2.846; 95%CI = 2.266–3.575; *p* < 0.001), ABSNW vs. NDNW (OR = 1.693; 95%CI = 1.309–2.189; *p* < 0.001), and ABSAW vs. NBSNW (OR = 4.613; 95%CI = 3.634–5.856; *p* < 0.001).

## 4. Discussion

The current study found that WC is more closely associated with hypogonadism than hyperglycemia in middle-aged men. To evaluate the associations between HbA1c and WC with hypogonadism, we applied Scheffe's posttest and compared the mean *T* values among the groups (Figure 1). The mean TT of group NBSAW ± was significantly lower than that of group ABSNW ±, and the mean TT of group ABSAW ± was significantly lower than that of group ABSNW ±, which indicates that the impact of WC on hypogonadism may be superior to the impact of HbA1c on hypogonadism. When we focus on Figure 2, we notice that the adjusted ORs show significant differences between NBSAW vs. NBSNW, ABSNW vs. NDNW, and ABSAW vs. NBSNW, which also indicates that an abnormal WC leads to a higher risk for HIS than does HbA1c.

A previous cross-sectional study that investigated associations of sex hormones and anthropometric markers in men and women from the general population found that TT was inversely associated with all anthropometric parameters and leptin in men, including WC [17]. That study is in line with other previous cross-sectional observational studies, which also revealed inverse associations between TT and multiple anthropometric markers in men, in particular with BMI and WC [18–20]. That is one of the reasons why we adjusted for BMI in Figure 2. WC was also reported to be inversely associated with total and free T, and WC was considered a useful tool in predicting lower T levels in men [21].

Furthermore, in recent decades, the validity of body mass index as an appropriate indicator of obesity has been questioned, and the most important limitation of body mass index is that it does not reflect regional body fat distribution. A previous systematic review and meta-analysis study indicated that waist circumference and other indices of central fatness that measures of central adiposity which represent visceral fat deposition could be used with body mass index as a supplementary approach to determine the risk of premature death [22]. The improved ability of waist circumference to predict health outcomes over BMI might be at least partially explained by the ability of waist circumference to identify adults with increased visceral adipose tissue mass [23]. Additionally, in a Chinese population-based study, we observed that the visceral adipose tissue dysfunction was significantly associated with lower total T levels after adjusting for age, smoking, neck and hip circumference, diabetes, and hypertension [24].

The association of low T with type 2 diabetes has been established in many studies. The two most famous large long-term studies, the MRFIT [25] and MMAS [26], suggest that low TT, FT, and SHBG are independent risk factors for the later development of type 2 diabetes. HbA1c was also strongly inversely associated with TT, with a previous study indicating that higher HbA1c levels lead to lower TT levels [14]. As previous studies have indicated, WC and HbA1c are both inversely associated with TT, but those studies did not determine which factor had a greater impact on hypogonadism.

WC is a more convenient parameter to measure than HbA1c. Thus, if we can prove that WC is better than HbA1c for predicting the risk of male hypogonadism, WC will be an easier method for detecting participants who may have a higher risk of hypogonadism in the future. After identifying such high-risk participants, earlier lifestyle interventions through exercise and diet control can offer this higher risk of hypogonadism group a chance to prevent the disadvantages associated with hypogonadism, such as a higher risk of metabolic syndrome [12], incident cardiovascular disease [13], and erectile dysfunction [14]. The question thus arises as to how abnormal WC and diabetes lead to hypogonadism. Kapoor et al. [15] found that T levels are inversely correlated with WC and diabetes, which is in line with our study. They noted that a plausible explanation for the inverse correlation of T levels with WC is the hypogonadal obesity cycle, which they expanded upon [27]. Specifically, visceral adipocytes have high aromatase activity, which converts T to estrogen. T inhibits the enzyme lipoprotein lipase, which takes up free fatty acids from adipocytes. Lower levels of T result in increased triglyceride levels in adipocytes, which promotes further adipocyte proliferation and hence higher aromatase activity. T levels are further lowered as a result of leptin resistance at the hypothalamic pituitary and testicular levels, causing reduced LH release and testosterone secretion. T levels are inversely correlated with WC, which is positively correlated with visceral adipose tissue. The nature of the relationship between diabetes and lower testosterone is unclear; however, men who have type 2 diabetes mellitus are more likely to have low serum T concentrations than nondiabetic men. In a review of 43 studies comprising 6,427 men, cross-sectional studies showed that men with type 2 diabetes had a mean serum T concentration that was 76 ng/dL lower than that of nondiabetic men. In the same review, longitudinal studies showed that men who had higher T concentrations had a lower risk of developing type 2 diabetes [28].

This study has some limitations, including its cross-sectional design. As such, despite observing an association between low T and abnormal HbA1c and WC values among the Asian population, the causality could not be clarified. Secondly, given the fact that the health examination of the MJ Health Screening Center is self-paid, we might have a population sample selection bias regarding income level. Finally, in the United Kingdom, which conducts a high percentage of immunoassays, considerable variation was noted in T measurements, and further work is required to achieve standardization [29].

## 5. Conclusions

The current study showed that WC should be the risk factor that is more closely associated with hypogonadism than hyperglycemia in middle-aged men.

## Figures and Tables

**Table 1:** Characteristics of the participants with or without hypogonadism.

	Without hypogonadism	With hypogonadism	*p*
	(*n* = 5081)	(*n* = 599)	
Age (yr)	45.9 ± 10.1	45.7 ± 9.5	0.627
WC (cm)	83.89 ± 8.62	90.91 ± 9.61	<0.001^∗∗∗^
BMI (kg/m^2^)	24.62 ± 3.30	27.64 ± 4.21	<0.001^∗∗∗^
HbA1c (%)	5.36 ± 0.70	5.65 ± 0.93	<0.001^∗∗∗^
FBS (mg/dl)	105.40 ± 19.11	113.96 ± 30.21	<0.001^∗∗∗^
TG (mg/dl)	135.05 ± 106.49	177.34 ± 113.48	<0.001^∗∗∗^
HDL (mg/dl)	52.20 ± 11.57	46.86 ± 9.37	<0.001^∗∗∗^
SBP (mmHg)	119.23 ± 14.67	124.19 ± 15.61	<0.001^∗∗∗^
DBP (mmHg)	77.58 ± 10.15	80.37 ± 10.48	<0.001^∗∗∗^
Total testosterone (ng/dl)	553.24 ± 205.73	242.17 ± 49.66	<0.001^∗∗∗^

WC: waist circumference (cm); BMI: body mass index (kg/m^2^); HbAlc: glycated hemoglobin (%); FBS: fasting blood sugar (mg/dl); TG: triglyceride (mg/dl); HDL: high-density lipoprotein cholesterol (mg/dl); SBP: systolic blood pressure (mmHg); DBP: diastolic blood pressure (mmHg). Comparisons between participants with and without hypogonadism were performed using an independent *T* test. ^∗^*p* < 0.05; ^∗∗^*p* < 0.01; ^∗∗∗^*p* < 0.001.

**Table 2:** The correlation analysis for HbAlc and WC in patients with and without hypogonadism.

	Without hypogonadism (*n* = 5081)	With hypogonadism (*n* = 599)	*p*
WC			
Normal			
(≤90 cm)	4013 (79.7)	329 (55.7)	
Abnormal			
(>90 cm)	1023 (20.3)	262 (44.3)	
HbAlc			
Normal			
(<5.7%)	3980 (78.3)	381 (63.6)	
Abnormal			
(≥5.7%)	1101 (21.7)	218 (36.4)	
Hba1c & WC			<0.00 l
NBSNW	3301 (65.5)	241 (40.8)	
NBSAW	640 (12.7)	133 (22.5)	
ABSNW	712 (14.1)	88 (14.9)	
ABSAW	383 (7.6)	129 (21.8)	

^∗^ *p* < 0.05; ^∗∗^*p* < 0.01; ^∗∗∗^*p* < 0.001.

**Table 3:** Logistic regression analysis for HbA1c or WC by stratified by WC or HbA1c.

	Stratified by WC						Stratified by HbA1c					
	Normal (≤90 cm)			Abnormal (>90 cm)			Normal (<5.7%)			Abnormal (≥5.7%)		
	OR^a^	95.0% C.I.		OR^a^	95.0% C.I.		OR^a^	95.0% C.I.		OR^a^	95.0% C.I.	
		Lower	Upper		Lower	Upper		Lower	Upper		Lower	Upper
HbA1c												
Normal	Ref.			Ref.								
Abnormal	1.433^∗^	1.088	1.887	1.473^∗∗^	1.106	1.962						
WC												
Normal							Ref.			Ref.		
Abnormal							1.591^∗∗∗^	1.230	2.058	1.83^7∗∗^	1.289	2.618

^∗^ *p* < 0.05; ^∗∗^*p* < 0.01; ^∗∗∗^*p* < 0.001. ^a^Model was applied for analyzing the relationship between with or without hypogonadism and in the HbA1c and WC groups after adjusting for BMI, TG, HDL, systolic blood pressure (SBP), and diastolic blood pressure (DBP).

## Data Availability

Data are available from the MJ Health Research Foundation. Due to the legal restrictions imposed by the government of Taiwan in relation to the “Personal Information Protection Act,” data cannot be made publicly available.
